# Whole-transcriptome sequencing and ceRNA interaction network of temporomandibular joint osteoarthritis

**DOI:** 10.3389/fgene.2022.962574

**Published:** 2022-10-05

**Authors:** Fan Wu, Yanxin An, Libo Zhou, Yuqing Zhao, Lei Chen, Jing Wang, Gaoyi Wu

**Affiliations:** ^1^ School of Basic Medicine, Heilongjiang Key Lab of Oral Biomedicine Materials and Clinical Application, Experimental Center for Stomatology Engineering, Jiamusi University, Jiamusi, China; ^2^ Department of Implantology, School of Stomatology, National Clinical Research Center for Oral Diseases & State Key Laboratory of Military Stomatology & Shaanxi Key Laboratory of Stomatology, Fourth Military Medical University, Xi’an, China; ^3^ Department of General Surgery, The First Affiliated Hospital of Xi’an Medical University, Xi’an, China; ^4^ School of Stomatology, Heilongjiang Key Lab of Oral Biomedicine Materials and Clinical Application, Experimental Center for Stomatology Engineering, Jiamusi University, Jiamusi, China; ^5^ Department of Orthodontics, School and Hospital of Stomatology, Cheeloo College of Medicine, Shandong University & Shandong Key Laboratory of Oral Tissue Regeneration & Shandong Engineering Laboratory for Dental Materials and Oral Tissue Regeneration, Shandong University, Jinan, China

**Keywords:** temporomandibular joint osteoarthritis, noncoding RNA, competitive endogenous RNA, high-throughput sequencing, interaction network

## Abstract

**Purpose:** The aim of this study was to conduct a comprehensive transcriptomic analysis to explore the potential biological functions of noncoding RNA (ncRNAs) in temporomandibular joint osteoarthritis (TMJOA).

**Methods:** Whole transcriptome sequencing was performed to identify differentially expressed genes (DEGs) profiles between the TMJOA and normal groups. The functions and pathways of the DEGs were analyzed using Metascape, and a competitive endogenous RNA (ceRNA) network was constructed using Cytoscape software.

**Results:** A total of 137 DEmRNAs, 65 DEmiRNAs, 132 DElncRNAs, and 29 DEcircRNAs were identified between the TMJOA and normal groups. Functional annotation of the DEmRNAs revealed that immune response and apoptosis are closely related to TMJOA and also suggested key signaling pathways related to TMJOA, including chronic depression and PPAR signaling pathways. We identified vital mRNAs, including *Klrk1, Adipoq, Cryab*, and *Hspa1b*. Notably, *Adipoq* expression in cartilage was significantly upregulated in TMJOA compared with normal groups (10-fold, *p* < 0.001). According to the functional analysis of DEmRNAs regulated by the ceRNA network, we found that ncRNAs are involved in the regulation of autophagy and apoptosis. In addition, significantly DEncRNAs (lncRNA-COX7A1, lncRNA-CHTOP, lncRNA-UFM1, ciRNA166 and circRNA1531) were verified, and among these, circRNA1531 (14.5-fold, *p* < 0.001) and lncRNA-CHTOP (14.8-fold, *p* < 0.001) were the most significantly downregulated ncRNAs.

**Conclusion:** This study showed the potential of lncRNAs, circRNAs, miRNAs, and mRNAs may as clinical biomarkers and provides transcriptomic insights into their functional roles in TMJOA. This study identified the transcriptomic signatures of mRNAs associated with immunity and apoptosis and the signatures of ncRNAs associated with autophagy and apoptosis and provides insight into ncRNAs in TMJOA.

## 1 Introduction

The temporomandibular joint (TMJ) is the only linkage joint in the human body that performs complex actions, such as opening, closing, chewing and speaking (Tamimi et al., 2018). Temporomandibular joint osteoarthritis (TMJOA) is a chronic progressive disease characterized by cartilage degradation, subchondral bone remodeling and osteophyte formation. The clinical symptoms of TMJOA include limited mandibular movement, impaired occlusal function and joint pain (Pinto et al., 2009; Schiffman et al., 2014), which seriously affect personal daily activities, psychosocial functions and quality of life. The incidence rate of TMJOA is 22%–38%, and the incidence of this disease is increasing (Peck et al., 2014). TMJOA is a multifactorial joint disorder that includes age, joint overload, trauma and psychological factors (Wang et al., 2015; Florjanski and Orzeszek 2021). At present, due to the lack of effective treatment methods (de Souza et al., 2012; Wang et al., 2015), it is necessary to further explore the unknown molecular mechanisms underlying TMJOA and discover potential biomarkers and new therapeutic targets.

Currently, an increasing number of scholars are paying attention to the roles of noncoding RNAs (ncRNAs) (particularly lncRNAs and circRNAs) in the occurrence and development of diseases, including tumors (Wang et al., 2019), periodontitis (Yu and Chi 2021), and osteoarthritis (Li et al., 2019). LncRNAs are RNA molecules with a length of more than 200 bases and lack protein-coding capacity (Marchese et al., 2017). It has been reported that lncRNAs can regulate gene expression through diverse mechanisms. CircRNAs are a type of RNA with a closed ring structure that is formed by RNA reverse splicing and exon or intron circularization (Memczak et al., 2013). CircRNAs are conserved, abundant and specifically expressed in certain tissues. Recent studies have revealed that lncRNAs and circRNAs participate in the initiation, progression and prognosis of TMJOA (Zhu et al., 2020; Zhu Y. et al., 2021). Exploring the roles of lncRNAs and circRNAs may facilitate important progress in the diagnosis and treatment of TMJOA.

Recent studies have described an intricate interplay among diverse RNA species. All RNA transcripts that contain miRNA-binding sites can communicate with and regulate each other by competing specifically for shared miRNAs and thus acting as competing endogenous RNAs (ceRNAs). The ceRNA hypothesis was first proposed by Professor PierPaolo Pandolfi in 2011 (Salmena et al., 2011). LncRNAs and circRNAs can act as ceRNAs or natural miRNA sponges and compete for binding to miRNAs to influence the expression of target genes (Hansen et al., 2013). CeRNA activity forms a large-scale and complex posttranscriptional regulatory network in the entire transcriptome, which greatly expands the functional genetic information in the genome (Smillie et al., 2018). Therefore, the study of ceRNA networks will open up new avenues for exploration of the molecular mechanism, diagnosis, and treatment of TMJOA in the future.

In a previous sequencing study of TMJOA samples, He et al, (2018) performed RNA-seq of condylar cartilage of rats. Condylar cartilage is the main lesion in TMJOA and can more effectively describe the pathological changes in TMJOA. Kang (2021) performed RNA-seq of peripheral blood mononuclear cells from humans. Inflammation and immune-related changes in peripheral blood mononuclear cells of TMJOA can indirectly reflect the pathological changes of TMJOA. Compared with condylar cartilage, peripheral blood samples are easier to obtain from humans, and thus, these samples can be feasibly used and have important clinical significance. However, the focus of these previous study was on transcriptome sequencing of mRNA. TMJOA is a complex regulatory network of interactions between diverse RNAs. Zhu et al, (2020) analyzed the expression profile of circRNAs in synovial tissues from human patients with TMJOA and established a ceRNA network by predicting the binding sites with miRNAs. Whole transcriptome sequencing of TMJOA was not performed in the abovementioned studies, and the construction of the ceRNA regulatory network of TMJOA remains to be explored. Therefore, we performed whole-transcriptome sequencing of the condylar cartilage of rats to obtain whole gene expression profiles and construct a ceRNA network to provide new insights for exploring the etiology of TMJOA.

In this study, high-throughput sequencing (HTS) was performed to determine the expression profiles of differentially expressed (DE) lncRNAs, circRNAs, miRNAs and mRNAs. Gene Ontology (GO) and Kyoto Encyclopedia of Genes and Genomes (KEGG) pathway enrichment analyses of the differentially expressed mRNAs were performed to detect the major functions of significant DE genes (DEGs). Furthermore, two ceRNA regulatory networks (a lncRNA‒miRNA-mRNA network and a circRNA-miRNA‒mRNA network) were established to explore the relationship between ncRNAs and mRNAs. The expression of several genes was validated. The functions of these genes need to be further studied to provide a reference for exploring the etiology of TMJOA.

## 2 Materials and methods

### 2.1 Animal model and sample collection

Female Sprague–Dawley (SD) rats (aged six weeks, weighing 160–180 g) were provided by the animal center of the Fourth Military Medical University, and the related experiments were approved by the ethics committee. Eighteen rats were divided into a control (CON) group and a unilateral anterior crossbite (UAC) group (Zhang et al., 2016). All procedures were performed under sodium pentobarbital anesthesia, and the animals were euthanized 8 weeks later. The condylar cartilage was carefully harvested, the blood and stains on the tissue surface were washed away with cold normal saline, and the samples were stored in liquid nitrogen until further use. Three samples were harvested from each group, and this process was repeated three times. The study was reviewed and approved by the Ethics Committee of Hospital of Stomatology of Shandong University.

### 2.2 Total RNA extraction and quality detection

Total RNA was isolated and purified using TRIzol reagent (Invitrogen, United States) following the manufacturer’s procedure. The RNA concentration and purity of each sample were quantified using a NanoDrop ND-1000 (NanoDrop, United States). The RNA integrity was assessed by an Agilent 2100 Bioanalyzer (Agilent Technologies, United States) with RIN >7. Ribosomal RNA was depleted from approximately 5 µg of total RNA using the Ribo-Zero™ rRNA Removal Kit (Norgen, Canada) according to the instructions.

### 2.3 Whole-transcript sequencing analysis and identification of DEGs

Library construction was performed following standard protocols, and sequencing was performed using the Illumina HiSeq 4000 platform by Lianchuan Biotechnology Co., Ltd. (Hangzhou, China). Cutadapt (http://cutadapt.readthedocs.org/en/stable/guide.html) was used to remove the reads that contained adaptor contamination, low-quality bases and undetermined bases. The sequence quality was then verified using FastQC (http://www.bioinformatics.babraham.ac.uk/projects/fastqc/). We used Bowtie2 and Hisat2 to map the reads to the genome of the samples. The mapped reads of each sample were assembled using StringTie. All the transcriptomes of the samples were then merged to reconstruct a comprehensive transcriptome using Perl scripts. After the final transcriptome was generated, StringTie and edgeR were used to estimate the expression levels of all the transcripts. StringTie was used to determine the expression levels of mRNAs, miRNAs, lncRNAs, and circRNAs by calculating FPKM values. The DEmRNAs, DEmiRNAs, DElncRNAs, and DEcircRNAs with log2 (fold change) > 1 or log2 (fold change) < -1 and with statistical significance (*p* value <0.05) were identified sing the R package edgeR.

### 2.4 Functional enrichment analysis of DEGs

GO analysis was performed for gene function annotation. Enrichment scores were calculated as the negative logarithm of the *p* value and used to determine the statistical significance of the GO term clusters targeted by the DE genes. KEGG analysis was performed to determine the biological pathways in which the genes in the network were involved, as quantified by the enrichment score.

### 2.5 Construction and analysis of the ceRNA network

Based on the ceRNA theory, we constructed a ceRNA regulatory network for the DEncRNAs and DEmRNAs to show the regulatory relationships among lncRNAs, circRNAs, miRNAs, and mRNAs. TargetScan (version 5.0) and miRanda (version 3.3a) were used to predict miRNA binding seed sequence sites, and the ceRNA network, which consisted of lncRNA‒miRNA pairs, circRNA-miRNA pairs, and miRNA‒mRNA pairs with the same miRNA nodes, was visualized using Cytoscape (version 3.7.0).

### 2.6 Enrichment analysis of target genes in the ceRNA network

Target genes in the ceRNA network were identified using the same approach as that used for the functional enrichment analysis of the DEGs.

### 2.7 Validation of the expression of candidate lncRNAs, circRNAs and mRNAs by real-time quantitative polymerase chain reaction (RT–qPCR)

We performed RT–qPCR to verify the partial sequencing results. TRIzol (Invitrogen, 15596026) was used to extract total RNA from the condylar cartilage tissues of the control group and UAC group. The RNA was reverse transcribed into cDNA using PrimeScript™ RT Master Mix (TaKaRa, RR036A). Master qPCR Mix (SYBR Green I) (Tsingke, T-TSE201) was used for the RT‒qPCR analysis (ABI, Prism^®^ 7500) according to the manufacturer’s recommended conditions. The target RNA and reference gene expression levels in the samples were measured by RT‒qPCR. The data were analyzed by the 2^-△△CT^ method. The sequences of the primers used are shown in Table 1.

**TABLE 1:** Primer sequences for RT-qPCR analysis of differentially expressed mRNA, lncRNA and circRNA levels.

Name	Primer type	Primer sequence
ADIPOQ	Forward	TGT​CTG​TAC​GAG​TGC​CAG​TG
	Reverse	CCC​GGT​ATC​CCA​TTG​TGA​CC
KLRK1	Forward	ACC​GTC​TCA​CAG​AAA​CAG​CC
	Reverse	AGG​CTC​CCC​TTG​TTT​TAC​CG
CRYAB	Forward	TGG​CTC​CAG​AGA​ACA​AGG​ATG
	Reverse	CAG​GGA​TTT​GGC​AGG​GTG​AC
HSPA1B	Forward	TTC​TGG​CTC​TCA​GGG​TGT​TG
	Reverse	AAC​GCA​AAG​AAC​ATG​CAA​CCT
NRF-1	Forward	TTA​CTC​TGC​TGT​GGC​TGA​TGG
	Reverse	CCT​CTG​ATG​CTT​GCG​TGG​TCT
LncRNA-COX7A1	Forward	GAG​TCT​GAA​GGA​AGC​AGC​GA
	Reverse	GAT​CAC​GCC​TGT​CCC​TAC​AC
LncRNA-CHTOP	Forward	GCA​TAG​GCA​GGT​GGT​TAT​CTG​T
	Reverse	TCG​TCA​AAG​GAG​AGT​CCA​AGT
LncRNA-UFM1	Forward	TTT​GAG​TAC​CAG​GCG​GTT​CC
	Reverse	ACG​CTG​AGG​ACT​TTG​TAC​GG
CircRNA166	Forward	CTC​TGA​GTA​AGC​GGC​AGA​GCC​T
	Reverse	ATT​GCT​CAG​AAG​CAC​AGA​ATC​ACT​T
CircRNA365	Forward	CAG​CAT​CAG​CCA​AAA​GTC​CCA​T
	Reverse	AGA​AGG​TCC​GGG​AAG​ATC​AAG​TC
CircRNA1531	Forward	TTA​AGC​AGA​AAG​GAG​TGA​TGA​ACC​C
	Reverse	AGT​GGC​ATG​TTG​CTG​GGT​AGA​TT

### 2.8 Statistical analysis

Statistical analyses were performed with the GraphPad Prism 8 (GraphPad, United States) and SPSS 23.0 software packages (SPSS, United States). All the values are expressed as the means ± standard errors of the mean; a *p* value <0.05 was considered to indicate statistical significance.

## 3 Results

### 3.1 Overview of the transcriptome profiling

**FIGURE 1:**
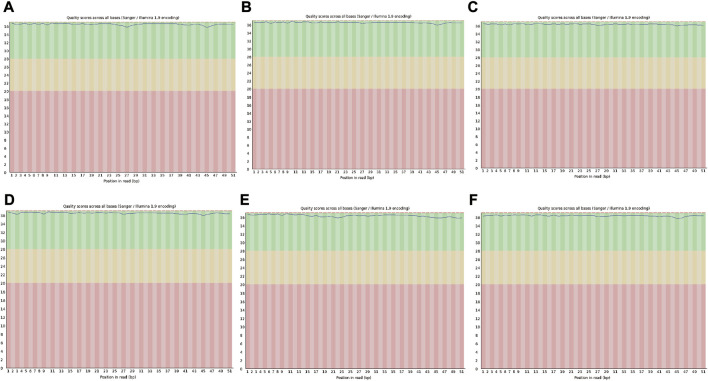
Quality evaluation of read lengths in sequencing files. **(A,B and C)** represent the CON group. **(D,E and F)** represent the UAC group. Proportion of bases with a quality value ≥ 30 (sequencing error rate of less than 0.001).

In view of the matrix degradation and cartilage degradation of TMJOA articular cartilage, the marker molecules *Col2al* and *Aggrecan* in the cartilage matrix were detected, and the expression of *Col2al* and *Aggrecan* was significantly decreased in the UAC group (Supplementary Figure S1). RNA-seq was then performed to analyze the whole transcriptome characteristics of TMJOA and normal group. Q Sanger (Q value), which was the value used to evaluate the sample quality, was greater than 30 for six samples, the sequencing quality was relatively high, and the probability of the sequencing equipment causing a base error at a certain position was less than 0.1% (Figure 1). An average of 87339322 raw reads were generated in six samples, and 81864520 clean reads remained after filtering the unqualified sequences with cut adapter; the proportion of clean reads was 93.73%. The detailed quality control results are listed in Table 2.

**TABLE 2:** Sequencing sequence statistics and quality control.

Sample	Raw data		Valid data		Valid Ratio (reads)	Q20%	Q30%	GC content%
	Read	Base (G)	Read	Base (G)				
CON1	92203214	13.83	86946982	13.04	94.30	99.97	97.84	48
CON2	85887700	12.88	81276116	12.19	94.63	99.97	97.95	48
CON3	91036064	13.66	85774768	12.87	94.22	99.97	97.97	48
UAC1	80126266	12.02	75879054	11.38	94.70	99.97	98.03	47
UAC2	94786056	14.22	87888716	13.18	92.72	99.97	98.03	48
UAC3	79996630	12.00	73421484	11.01	91.78	99.97	98.13	47

CON, control; UAC, unilateral anterior crossbite.

### 3.2 Analysis of DEGs by RNA-seq

**FIGURE 2:**
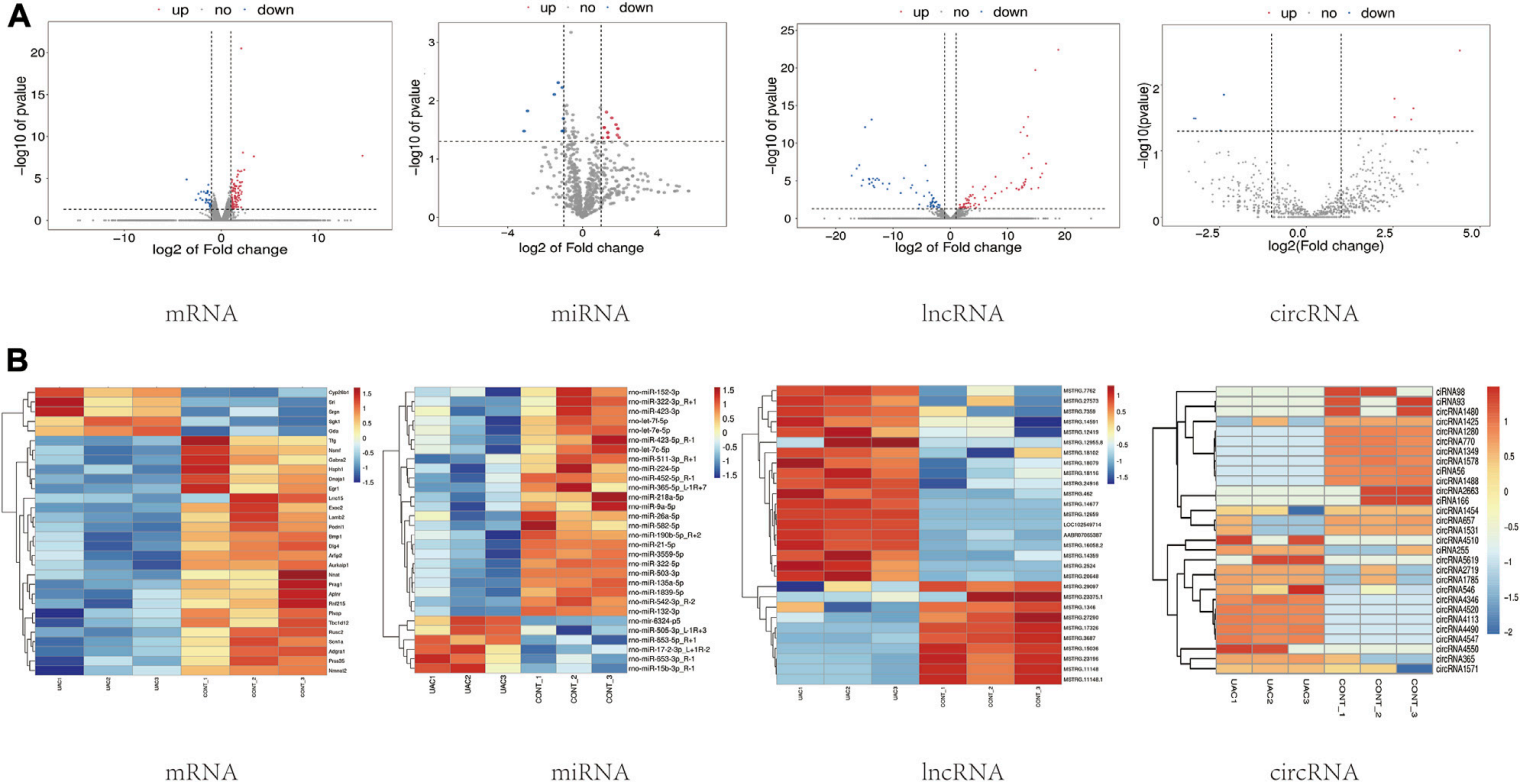
**(A)** Visual statistical results of differential gene analysis. Volcano plots of mRNA, miRNA, lncRNA, circRNA expression. Each dot in the graph represents a specific gene or transcript; the red dots represent significantly upregulated genes; the blue dots represent significantly downregulated genes, and the gray dots represent genes without significantly different expression. **(B)** Cluster analysis (heatmaps). Red and blue indicate up- and downregulation, respectively. “CONT” represents healthy controls, and “UAC” represents the temporomandibular joint osteoarthritis model.

Through high-throughput sequencing and associated bioinformatics analysis, we identified 137 DEmRNAs (112 upregulated and 35 downregulated), 65 DEmiRNAs (27 upregulated and 38 downregulated), 132 DElncRNAs (77 upregulated and 55 downregulated), and 29 DEcircRNAs (14 upregulated and 15 downregulated). The overall distribution of DEGs can be observed by generating a volcano plot. Figure 2A shows the distribution of all the significantly DEmRNAs, DEmiRNAs, DElncRNAs, and DEcircRNAs in two dimensions (−log_10_ *p* value and log_2_ (fold change, FC)) in a volcano plot.

### 3.3 Cluster analysis of RNA-seq data

To preliminarily explore the phenotypic differences associated with the different transcriptomes in normal and TMJOA cartilage, a heatmap was generated after z-conversion of the relative abundance values of the DEmiRNAs, DEmRNAs, DElncRNAs and DEcircRNAs. In the cluster analysis diagram, the trends in gene expression in the samples can be intuitively observed according to the color changes (log^2^FC ≥ 1; *p* value <0.05) (Figure 2B). Hierarchical clustering showed that the lncRNAs, circRNAs, miRNAs, and mRNAs were well distinguished between TMJOA patients and healthy controls. Obvious differences in the DEGs profiles were found between the two groups. The results from the cluster analysis were similar to the DEGs filtering results.

### 3.4 Functional enrichment analysis of DEGs

**FIGURE 3:**
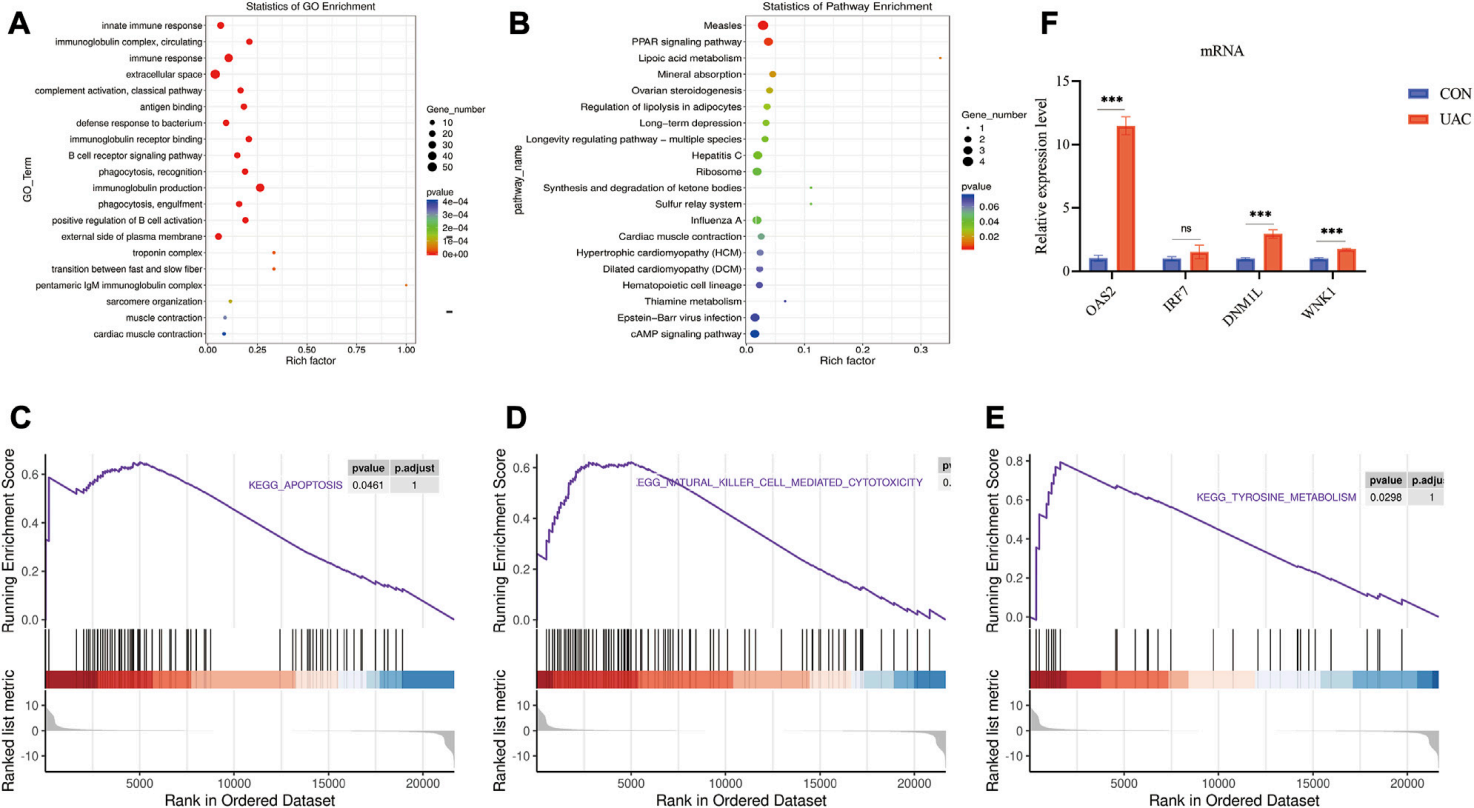
**(A)** show some of the significantly enriched GO terms associated with the mRNA target genes. **(B)** shows some of the significantly enriched pathways associated with the mRNA target genes. The size of the spot indicates the number of genes enriched in the pathway, and the color of the spot indicates the significance level of the enriched pathway. **(C,D and E)** are gene set enrichment analysis (GSEA) results. **C** presents the tyrosine metabolism results. **D** presents the natural killer cell-mediated cytotoxicity results. E presents the apoptosis results. (F) qRT-PCR validation of the essential genes of enrichment analysis including *Klrk1, Adipoq, Cryab, Hspa1b.* **p < 0.01.

The functions of lncRNAs, circRNAs, and miRNAs are mainly to regulate the expression of mRNAs. Therefore, we performed GO and KEGG analyses using Metascape software to assess the molecular functions of the DEmRNAs to reveal the roles of the DElncRNAs, DEcircRNAs, and DEmiRNAs. The top 20 GO items are shown in Figure 3A. The top GO terms enriched in the upregulated mRNAs were innate immune response, immune response, extracellular space, and defense response to bacterium. The top GO terms enriched in the downregulated mRNAs were muscle contraction and transition between fast and slow fibers. In addition to the basic cellular activities, immune-related biological processes, such as phagocytosis, recognition, complement activation, classical pathway, and immunoglobulin production, were also identified from the GO enrichment analysis, particularly from the analysis of the upregulated genes (Supplementary Table S1).

The top KEGG pathways enriched in the differentially expressed mRNAs included the PPAR signaling pathway, mineral absorption, and long-term depression (Supplementary Table S2). The top 20 enriched KEGG pathways are shown in Figure 3B. To further explore the enrichment analysis of DEGs in intact and damaged cartilage, we analyzed the underlying molecular functions by GSEA and showed that these genes are closely associated with apoptosis, natural killer cell-mediated cytotoxicity, and tyrosine metabolism (Figures 3C–E). Immune response-related genes (*Klrk1*), PPAR signaling pathway-related genes (*Adipoq*), and apoptosis pathway-related genes (*Cryab* and *Hspa1b*) were selected for further verification by qRT‒PCR. The expression levels of *Klrk1* and *Adipoq* were upregulated in TMJOA, and the expression levels of *Cryab* and *Hspa1b* were downregulated.

### 3.5 Construction of the ceRNA network

**FIGURE 4:**
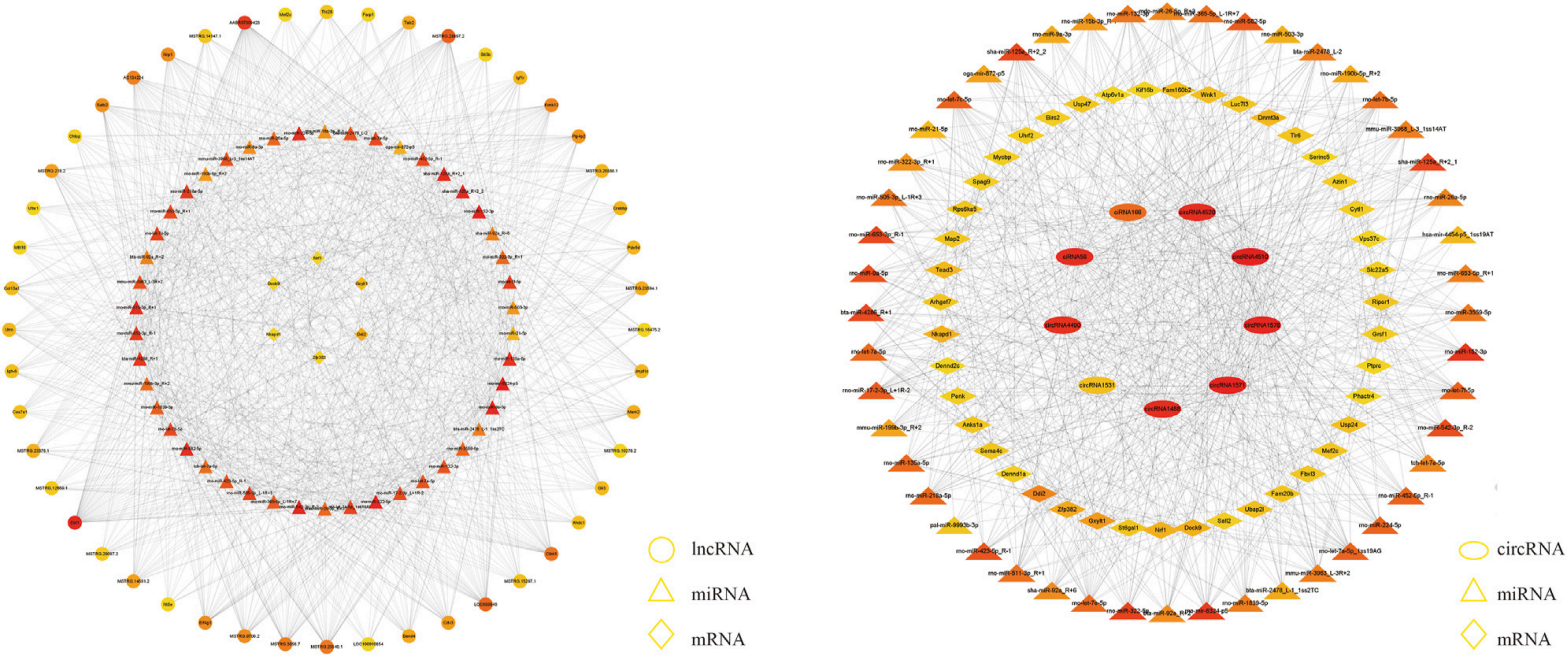
A The lncRNA-miRNA-hub mRNA network. In this figure, lncRNAs, miRNAs, and mRNAs are indicated by circles, triangles, and diamonds, respectively. The darker red color of a circle means that the gene has a higher degree and has more connections with other genes; conversely, the closer a circle color is to yellow, the lower the degree of the gene is, and the fewer connections it has with other genes. B The circRNA-miRNA-hub mRNA network. In this figure, circRNAs, miRNAs, and mRNAs are indicated by ellipses, triangles, and diamonds, respectively.

**FIGURE 5:**
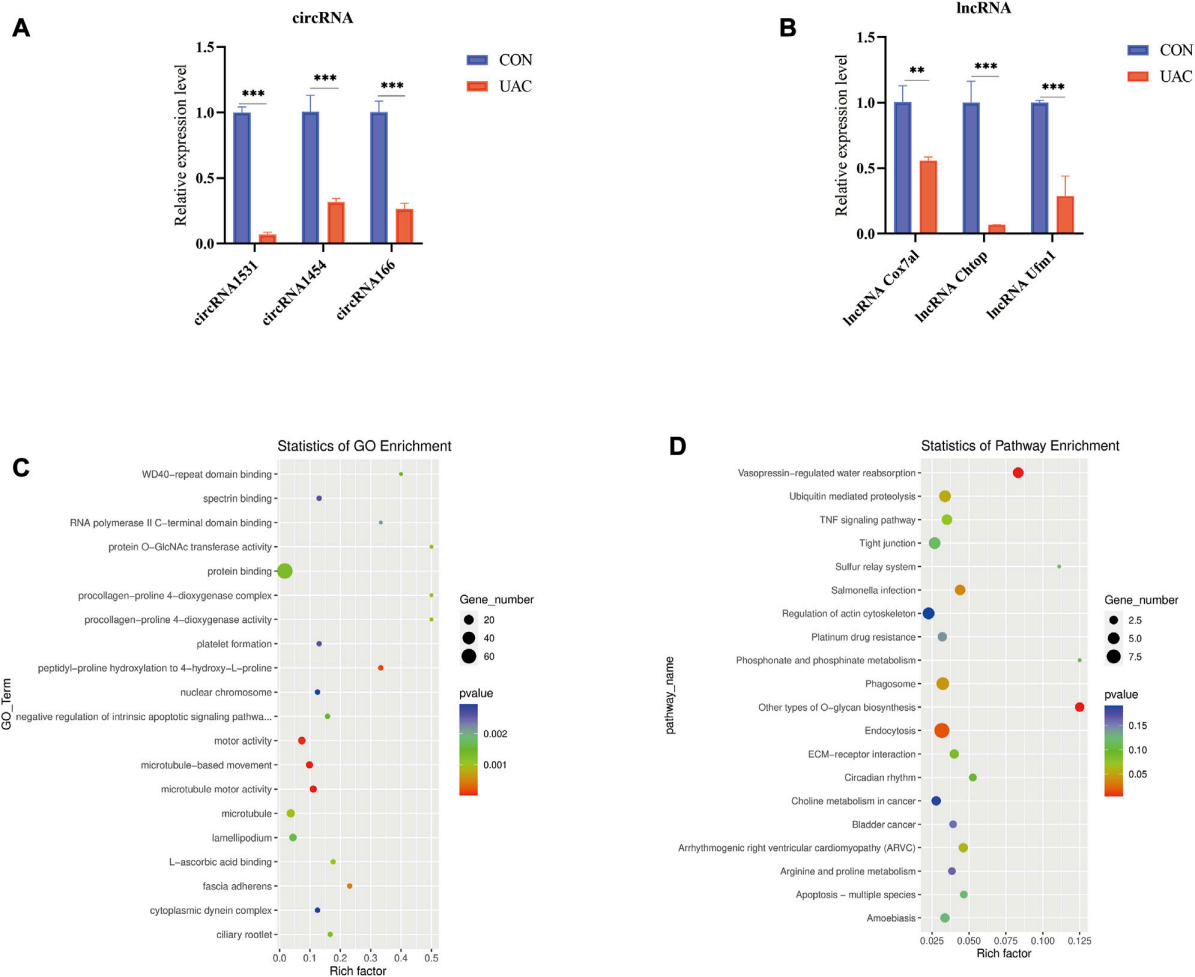
**(A and B)** qRT-PCR was used to detect the expression of lncRNA and circRNA in ceRNA network. **(C)** show some of the significantly enriched GO terms of the target genes. **(D)** shows some of the enriched pathways of the target genes. The size of the spot indicates the number of genes enriched in the pathway, and the color of the spot indicates the significance level of the enriched pathway.

We explored the functions of ncRNAs by constructing a ceRNA network. The lncRNA‒miRNA-mRNA ceRNA network was composed of 48 lncRNAs, 46 miRNAs and six mRNAs (Figure 4A). The circRNA-miRNA‒mRNA ceRNA network was composed of nine circRNAs, 43 miRNAs and 48 mRNAs (Figure 4B). LncRNAs/circRNAs can regulate one or more mRNAs in the ceRNA network. For example, lncRNA-CHTOP targeted *Nrf1*, *Ddi2*, and *Dock9*, and ciRNA166 regulated *Dnmt3A*, *Ddi2*, and *Dock9* (Supplementary Table S3). As differential factors associated with TMJOA, these genes play important roles in the disease process. We focused on lncRNAs and circRNAs involved in ceRNA network regulation, namely, lncRNA-COX7A1, lncRNA-CHTOP, lncRNA-UFM1, ciRNA166, circRNA365, and circRNA1531, which were selected for further verification through RT‒qPCR. In addition to circRNA365, seven out of eight were validated. The expression levels of lncRNA-COX7A1, lncRNA-CHTOP, lncRNA-UFM1, ciRNA166, and circRNA1531 were significantly downregulated, which was consistent with the RNA sequencing results (Figures 5A,B).

### 3.6 Enrichment analysis of target genes in the ceRNA network

To better understand the mechanisms involved in TMJOA, we performed GO enrichment and KEGG pathway analyses of the target mRNAs of the ceRNA network. The functions of GO enrichment in the ceRNA network were as follows: regulation of autophagy of mitochondria, positive regulation of the extrinsic apoptotic signaling pathway, positive regulation of the canonical Wnt signaling pathway (Figure 5C). The KEGG enrichment pathways included endocytosis, Salmonella infection, phagosome, and ubiquitin-mediated proteolysis. The enrichment analysis results suggested that apoptosis may be the main mechanism underlying TMJOA pathogenesis (Figure 5D, Supplementary Tables S4, S5).

## 4 Discussion

To better understand the global gene expression patterns and the potential regulatory mechanisms of TMJOA, a comprehensive analysis of the transcriptome based on high-throughput sequencing technology was performed. In this study, we performed HTS on three groups of normal and inflamed condylar cartilage samples to analyze differential gene expression profiles, and the results revealed 137 DEmRNAs, 65 DEmiRNAs, 132 DElncRNAs, and 29 DEcircRNAs. GO, KEGG and GSEA enrichment analyses revealed that the DEmRNAs were mainly involved in the immune response, PPAR signaling pathway and apoptosis. The regulatory roles of the DElncRNAs and DEcircRNAs in TMJOA were analyzed by a ceRNA interaction network. Then, we selected some key mRNAs, lncRNAs, and circRNAs for RT–qPCR verification, and the results were consistent with the sequencing results.

The biological functions and potential pathways related to the mRNAs were analyzed by GO and KEGG enrichment analyses. Notably, we found that the significantly enriched functions were closely related to the immune response, including the immune response, immunoglobulin production, and natural killer cell-mediated cytotoxicity. These results indicate that immune-related molecular functions and biological processes participate in the pathogenesis of TMJOA. This finding is consistent with reports in the literature showing that OA is closely related to the immune system (Haseeb and Haqqi 2013). Moreover, a study on the transcriptome of the peripheral blood of young females confirmed that the immune regulatory response participates in the pathogenesis of TMJOA (Kang 2021). Our study showed that the key molecules *Klrk1*, *Oas2* and *Irf7* exhibit large changes in expression levels, and the expression of these molecules were increased by 2-3fold, which indicates they may be involvement in the pathogenesis of TMJOA. *Klrk1* gene polymorphisms lead to infectious diseases, cancer, and autoimmune diseases (Lanier 2015). RT‒qPCR verified the high expression of *Klrk1* in TMJOA. Andersson et al, (2011) showed that blocking *Klrk1* improves the collagen-induced arthritis. In addition, the expression of *Oas2* and *Irf7* was increased in TMJOA (Supplementary Figure S2). Three studies recently showed that *Oas2* is highly expressed in RA based on high-throughput sequencing and bioinformatics, and *Oas2* may be a gene signature in RA development *via* regulation of the immune response (van Baarsen et al., 2010; He et al., 2016; Fang et al., 2021). Some studies found that *Irf7* is closely related to RA and its expression is elevated in RA. (Park et al., 2008; Smith et al., 2013; Mariaselvam et al., 2017). However, the current research on these key molecules has mainly focused on the pathogenesis and treatment of RA. The function of these molecules in TMJOA remain to be studied in depth.

KEGG enrichment analysis indicated that the PPAR signaling pathway was significantly enriched in key pathways. *Adipoq* is the downstream target gene of PPAR signaling pathway. In our study, the *Adipoq* was significantly enriched in the PPAR pathway, and its expression was significantly elevated in TMJOA. A previous study found that the RS1501299 polymorphism of the *Adipoq* increases the risk of knee osteoarthritis (Shang et al., 2019), and the interaction between the rs1501299 (Adipoq) and rs662 (Pon1) gene polymorphisms may play an important role in the development of osteoarthritis (Fernandez-Torres et al., 2019). Mutation of the *Adipoq* may be associated with an increased risk of TMJOA. Notably, long-term depression is also a significantly enriched pathway. Psychological factors reportedly influence the onset of TMJ disorders, the course of the disease and the response to treatment (Florjanski and Orzeszek 2021).

In addition, GSEA showed that apoptosis occurred during TMJOA. At present, many studies have focused on the interaction between apoptosis and OA (Kim and Blanco 2007; Musumeci et al., 2015). However, the relative role of chondrocyte apoptosis in the pathogenesis of OA is difficult to evaluate. We identified genes involved in apoptosis-related processes, including the genes Cryab and Hspa1b, whose expression was downregulated, and the genes Cd5, Nr4a3, Ifit3, and Klrk1, whose expression was upregulated. The results suggested that these genes are related to the occurrence and development of TMJOA. Moreover, our results have been confirmed by related studies (Nielsen et al., 2014; Bao et al., 2019; Ma et al., 2020; Yokoyama-Kokuryo et al., 2020).

Few studies have investigated the transcriptional regulation of microRNAs in TMJOA. In this study, we identified some important DEmiRNAs associated with TMJOA. To name a few, the expression levels of rno-miR-653–5p and rno-miR-15b-3p were upregulated, and those of rno-miR-135a-5p, rno-miR-132–3p, rno-miR-9a-5p, rno-miR-152–3p, and rno-miR-26a-5p were downregulated. In addition, these molecules also participate in the regulation of the ceRNA network. Among these molecules, miR-15b-3p and miR-26a-5p have been confirmed by previous studies to be related to osteoarthritis. Cao et al, (2019) showed that inhibition of miR-15b expression contributes to enhanced proliferation and reduced apoptosis of chondrocytes through increases in *Igf1, Igf1r*, and *Bcl2* expression. Rasheed et al, (2016) reported that miR-26a-5p is a direct regulator of *iNOS* expression in human chondrocytes. The mechanisms through which other miRNAs function in TMJOA remain to be studied.

Currently, accumulating evidence suggests that ncRNAs, particularly lncRNAs and circRNAs, play important roles in the progression of TMJOA. The alterations in the expression of lncRNAs and circRNAs in TMJOA may result in the aberrant expression of target genes. Therefore, we constructed ceRNA networks to further research the regulatory roles of DElncRNAs and DEcircRNAs in TMJOA. Understanding the intricate interplay among diverse ceRNA networks will lead to significant insight into gene regulatory networks and will have implications in TMJOA treatment. Xu et al, (2020) revealed that Pvt1 upregulates *Tnf-α* in synoviocytes by sponging miR-211–3p and induces chondrocyte apoptosis. Zhu Y. et al, (2021) suggested that lncRNA XIST modulates SMSC chondrogenic differentiation *via* the miR-27b-3p/Adamts-5 axis. In this study, we found that lncRNAs and circRNAs in the ceRNA network are mainly involved in the progression of TMJOA by regulating chondrocyte apoptosis and autophagy. In the lncRNA-ceRNA network, lncRNA-COX7A1 acts as a sponge for miR-6324–5p to regulate the target gene *Nrf1*, and lncRNA-CHTOP acts as a sponge for miR-653–3p to regulate *Nrf1*. Several studies have confirmed that *Nrf1* deficiency plays a key role in the inflammatory disease predisposition and immune disease because it exerts negative effects on the proliferation, survival and function of cells (Chong et al., 2020; Shen et al., 2021). For example, Oh et al, 2012) previously demonstrated that hepatocyte-specific deletion of *Nrf1* in mice results in spontaneous apoptosis, inflammation, and liver tumor development. By exploring the DEmiRNA-DEmRNA relationships, we found that *Nrf1* could bind to many miRNAs and that its expression was negatively correlated with that of these miRNAs. Our study showed that rno-miR-363–3p expression was upregulated and that *Nrf1* expression was downregulated. *Nrf1* may be a potential target gene of miR-363–3p. This finding is consistent with the results reported by Zhang et al, (2020). In rats with OA and LPS-treated chondrocytes, inhibiting the expression of miR-363–3p increases the expression of *Nrf1* and prevents chondrocyte apoptosis. According to the ceRNA network of lncRNAs, lncRNAs play a role in the pathogenesis of TMJOA through the indirect regulation of *Nrf1* expression. However, further experiments are needed to determine its function.

CircRNAs can also be combined with miRNAs alone to regulate downstream target genes. Hu et al, (2019) reported that hsa_circ_0000448 upregulation in synovial tissues of TMJOA is involved in the TNF-α signaling pathway through a ceRNA mechanism by targeting related microRNAs. Zhu et al, (2020) reported that circGCN1L1 induces inflammation in TMJ synoviocytes and decreases anabolism of the extracellular matrix through miR-330–3p and TNF-α. Our ceRNA network revealed that ciRNA166 and circRNA1531 can combine with miR-9a-5p to regulate the target gene Dnmt3a, which plays an important regulatory role in apoptosis, ECM degradation, and chondrocyte injury in osteoarthritis (Ma et al., 2019; Zhu H. et al., 2021; Zhang et al., 2021). Moreover, the indirect regulation of Dnmt3a by circRNAs has been observed in studies of OA. Zhang et al, (2021) revealed that circ_SEC24A expression is upregulated in osteoarthritic cartilage tissues and IL-1β-treated chondrocytes, and this upregulation is accompanied by miR-26b-5p downregulation and Dnmt3a upregulation. Zhu H. et al, (2021) demonstrated that circ-136474 downregulation and miR-766–3p upregulation exert chondroprotective effects against IL-1β-induced oxidative stress by suppressing Dnmt3a expression. Combined with the results from our enrichment analyses, these results suggest that the circRNA166/miR-9a-5p/Dnmt3a and circRNA1531/miR-9a-5p/Dnmt3a networks may regulate chondrocyte apoptosis, ECM degradation, and chondrocyte injury in the occurrence and development of TMJOA.

Most of the lncRNAs and circRNAs identified in our study have not yet been studied, and we thus analyzed the functions of DEncRNAs using the targeted mRNAs. In our study, GO analysis of the target genes revealed the significantly enriched biological functions, including regulation of autophagy of mitochondria, positive regulation of the extrinsic apoptotic signaling pathway, and regulation of the Wnt signaling pathway. It has been confirmed that the regulation of autophagy in mitochondria is closely related to TMJOA. Mitophagy impairment gives rise to the progressive accumulation of defective mitochondria, which leads to ECM degradation and contributes to the degeneration of cartilage (Sun et al., 2021). In this study, Dnm1l was found to be the enriched gene in the regulation of autophagy of mitochondria. The loss of Dnm1l resulted in increased oxidative damage in mitochondria (Zhang et al., 2012). However, the role of Dnm1l in TMJOA remains to be investigated. The Wnt signaling pathway is an important pathway in TMJOA that regulates chondrocyte apoptosis (Liu et al., 2019), cartilage degradation (Held et al., 2018) and the inflammatory response (Liu et al., 2021). It has been reported that inhibition of the Wnt signaling pathway can reduce the severity of osteoarthritis (Lietman et al., 2018). Yang et al. (Yang et al., 2020) reported that lncRNA HOTAIR promotes cartilage degradation in osteoarthritis by activating the Wnt signaling pathway. Our study revealed the involvement of Wnk1 in regulation of the Wnt signaling pathway. The ceRNA network suggested that ciRNA166 may promote TMJOA progression by regulating miR-26a-5p/wnk1, but further research is needed. KEGG analysis of the target genes showed that endocytosis, salmonella infection, phagosome and ubiquitin-mediated proteolysis are involved in TMJOA. Previous studies have suggested that the biological process of ubiquitination leads to OA by regulating the expression of inflammatory cytokines (Radwan et al., 2015; Ge et al., 2021). Zucker-Franklin (1966) showed that phagosomes participate in the regulation of synovial fluid leukocytes during RA development. Endocytosis, infection and phagosomes have not been studied in TMJOA. GO and KEGG enrichment analyses of the target genes may provide a new idea for exploring the mechanism of TMJOA.

However, this study has some limitations. To determine the direct pathological cause of cartilage damage, we extracted condylar cartilage rather than other tissues or blood for whole-transcriptome sequencing. The surface of the TMJ is fibrocartilage and subchondral bone, which are sensitive to stress. Mechanical stimulation is considered the key factor in the development of TMJOA. The UAC model we selected is a well-developed model that is used to establish TMJOA, and this model provides a better approach for modeling abnormal mechanical stimulation. There are many modeling methods, and the differences in the sequencing results from different models need to be verified in subsequent experiments. Due to the large amount of information obtained from the sequencing results, we did not verify the expression levels of all the DEGs. In addition, this study is only a preliminary exploration, and the results will be verified by subsequent experiments.

In our study, we found several genes that may play an important role in the occurrence and development of TMJOA. Further studies using various knockdown or knock-in strategies to test the functional roles of these genes are needed to better understand their role in TMJOA. The ceRNA network suggested underlying molecular mechanisms, such as the lncRNAs Chtop/miR-653–3p/Nrf1, circRNA1531/miR-9a-5p/Dnmt3a, and miR-26a-5p/wnk1, which may play important regulatory roles in TMJOA. Further studies using various knockdown or knock-in strategies and luciferase assays or PCR assays were performed to verify these mechanisms. In addition, the ceRNA mechanisms need to be further elucidated by *in vivo* experiments to provide more evidence.

## 5 Conclusion

In this study, we identified the expression profiles of DElncRNAs, DEcircRNAs, DEmiRNAs and DEmRNAs that may be associated with TMJOA progression. We found that the immune response, apoptosis, depression, and PPAR signaling pathways are closely related to TMJOA development. In addition, this study constitutes the first exploration of the potential functions of ncRNAs in TMJOA by constructing a ceRNA network using circRNAs and lncRNAs. Our study provides novel insight into TMJOA based on the ceRNA regulatory network. However, the roles of lncRNAs and circRNAs in TMJOA development need to be further studied and confirmed *in vivo* and *in vitro*.

## Data Availability

The data presented in the study are deposited in the GEO repository, accession number GSE207463.
