# Supplementary figures and images for: Whole-transcriptome sequencing and ceRNA interaction network of temporomandibular joint osteoarthritis
Source: Front Genet. 2022 Oct 5;13:962574. doi: 10.3389/fgene.2022.962574 (PMC9581126; doi:10.3389/fgene.2022.962574)

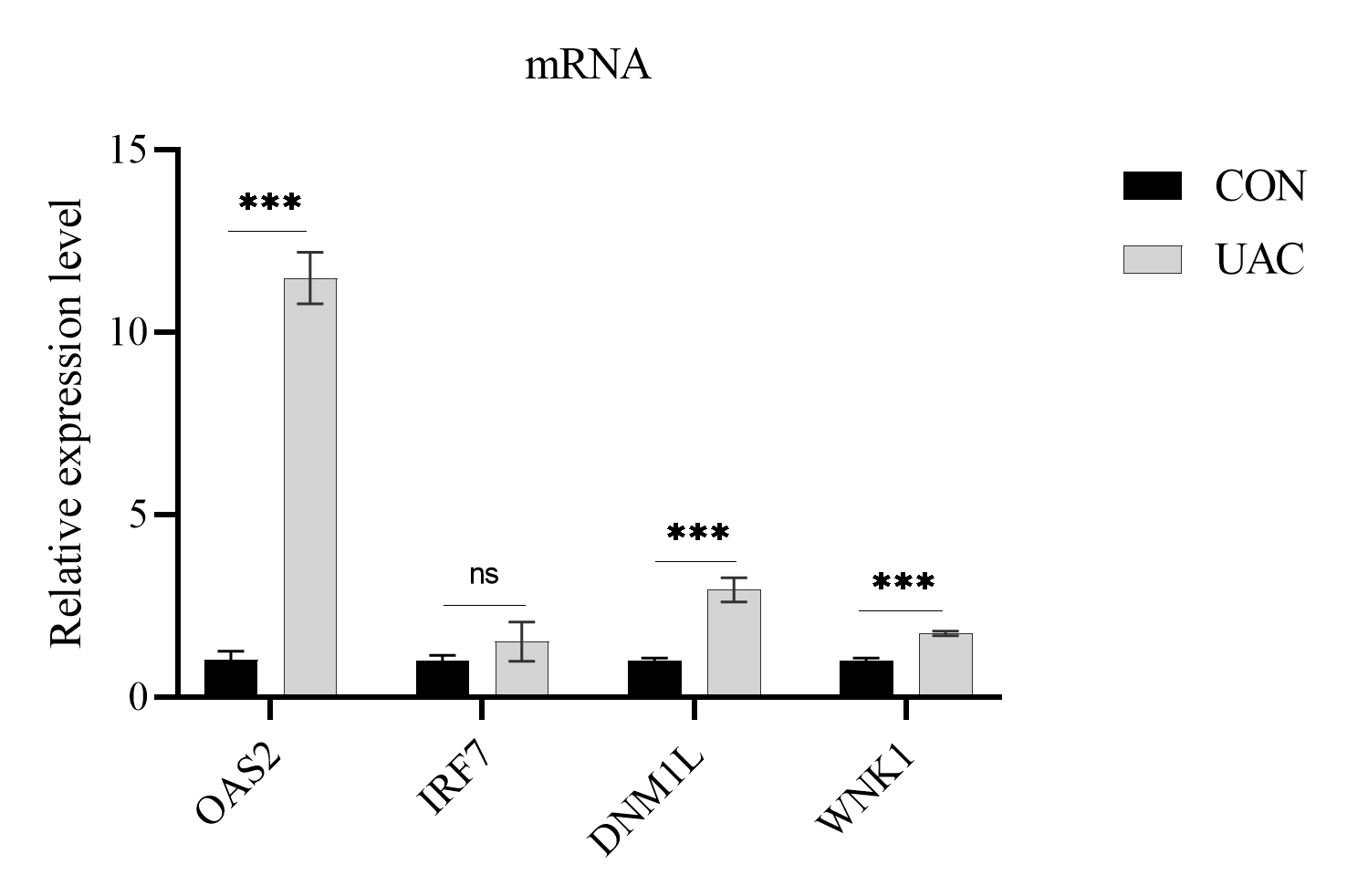

Supplement: Supplementary file 3 [file Image2.TIF]

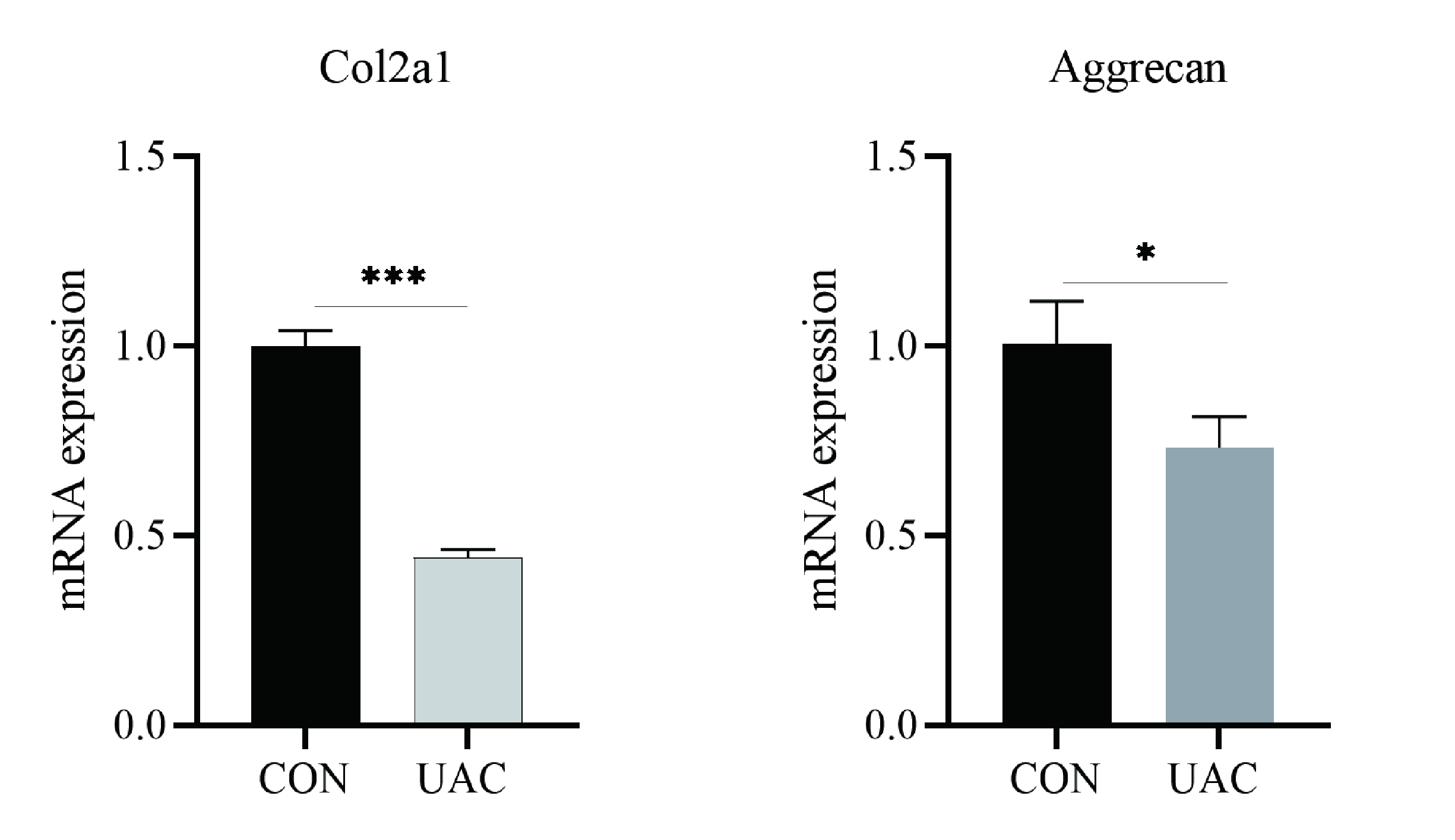

Supplement: Supplementary file 4 [file Image1.TIF]
